# The economic impact of prevention, monitoring and treatment strategies for iodine deficiency disorders in Germany

**DOI:** 10.1530/EC-20-0384

**Published:** 2020-11-27

**Authors:** Monika Schaffner, Ursula Rochau, Nikolai Mühlberger, Annette Conrads-Frank, Vjollca Qerimi Rushaj, Gaby Sroczynski, Eftychia Koukkou, Betina Heinsbaek Thuesen, Henry Völzke, Wilhelm Oberaigner, Uwe Siebert

**Affiliations:** 1 Department of Public Health, Health Services Research and Health Technology Assessment, Institute of Public Health, Medical Decision Making and Health Technology Assessment, UMIT – University for Health Sciences, Medical Informatics and Technology, Hall i.T., Austria; 2 Faculty of Pharmacy, School of PhD Studies, Ss. Cyril and Methodius University, Skopje, Macedonia; 3 General Hospital ‘Elena Venizelou’, Athens, Greece; 4 Centre for Clinical Research and Prevention, Capital Region of Denmark, Denmark; 5 Institute for Community Medicine, University Medicine Greifswald, Greifswald, Germany; 6 Center for Health Decision Science, Department of Health Policy and Management, Harvard Chan School of Public Health, Boston, Massachusetts, USA; 7 Department of Radiology, Institute for Technology Assessment, Massachusetts General Hospital, Harvard Medical School, Boston, Massachusetts, USA

**Keywords:** cost analysis, resource analysis, prevention program, iodine deficiency, thyroid disorders, salt iodization

## Abstract

**Objective:**

More than 30% of the German population suffers from mild to moderate iodine deficiency causing goiter and other iodine deficiency disorders (IDDs). The economic burden of iodine deficiency is still unclear. We aimed to assess costs for prevention, monitoring and treatment of IDDs in Germany.

**Design:**

We performed a comprehensive cost analysis.

**Methods:**

We assessed direct medical costs and direct non-medical costs for inpatient and outpatient care of IDDs and costs for productivity loss due to the absence of work in 2018. Additionally, we calculated total costs for an IDD prevention program comprising universal salt iodization (USI). We performed threshold analyses projecting how many cases of IDDs or related treatments would need to be avoided for USI to be cost-saving.

**Results:**

Annual average costs per case in the year of diagnosis were € 211 for goiter/thyroid nodules; € 308 for hyperthyroidism; and € 274 for hypothyroidism. Average one-time costs for thyroidectomy were € 4184 and € 3118 for radioiodine therapy. Average costs for one case of spontaneous abortion were € 916. Annual costs of intellectual disability were € 14,202. In the German population, total annual costs for USI would amount to 8 million Euro. To be cost-saving, USI would need to prevent, for example, 37,900 cases of goiter/thyroid nodules.

**Conclusion:**

USI potentially saves costs, if a minimum amount of IDDs per year could be avoided. In order to recommend the implementation of USI, a full health-economic evaluation including a comprehensive benefit-harm assessment is needed.

## Introduction

Iodine deficiency is one of the world’s most common nutritional health problems. It causes thyroid disorders, such as goiter, hypothyroidism and hyperthyroidism and a variety of other iodine deficiency disorders (IDDs). Worldwide, about 2 billion people suffer from iodine deficiency and its consequences (1). According to the World Health Organization (WHO), more than 390 million people in Europe are affected (2).

Although Germany is no longer a designated iodine-deficient area according to the WHO, two recent epidemiological studies in Germany revealed that about 30% of children and 29% of adults are still considered to be mildl to moderately iodine deficient (3, 4, 5). According to the ‘Arbeitskreis Jodmangel’ – a multi-sectoral working group – about 40,000 Germans have to undergo radioiodine therapy each year. In addition, there are almost 90,000 Germans who have to undergo thyroidectomy (6). It is estimated that diagnosis and treatment of endemic goiter due to iodine deficiency cause annual healthcare costs of more than one billion Euros in Germany (7).

Universal salt iodization (USI) programs are commonly used to prevent iodine deficiency and its consequences. In the year 1994, the WHO and UNICEF Joint Committee on Health Policy recommended the fortification of household salt with small amounts of potassium iodate as a safe and sustainable way to prevent iodine deficiency (8). However, the financial burden of iodine deficiency and the cost-effectiveness of IDD prevention programs are still unclear.

Evidence-based approaches are needed for a systematic evaluation of long-term effectiveness and cost-effectiveness of health care interventions. Decision-analytic models can be used to inform decision-makers about the value of health care technologies including benefits, risks and costs (9, 10). This approach allows synthesizing the current evidence from multiple sources to support decision making under uncertainty and consider multiple outcomes relevant for patients and society such as quality-adjusted life years (QALY) and costs (10, 11). Cost analyses provide an important basis for such assessments and are therefore an essential method used in health technology assessment.

The purpose of our study was to perform a comprehensive cost analysis to assess costs for prevention, monitoring and treatment of IDDs in Germany. Additionally we aimed to estimate how many cases of IDDs or related treatments would need to be avoided for an IDD prevention program to be cost-saving.

## Materials and methods

Our analysis includes two parts. In the first part, we assessed resource utilization and costs disaggregated by different items as well as a total cost per IDD per patient. In the second part, we performed threshold analyses projecting how many cases of IDDs or related treatments would need to be avoided for an IDD prevention program to be cost-saving.

In general, we followed guidelines from the Institute for Quality and Efficiency in Health Care (IQWIG) (‘Institut für Qualität und Wirtschaftlichkeit im Gesundheitswesen’) for health economic evaluations (12) as well as international guidelines (13). We considered resource utilization items from the impact inventory of the Second Panel on Cost-Effectiveness in Health and Medicine (14, 15). For reporting economic results, we followed the Consolidated Health Economic Evaluation Reporting Standards (CHEERS) Statement (16).

We evaluated the costs associated with the prevention, diagnosis, treatment and inpatient and outpatient care of the following IDDs from the German societal perspective: hyperthyroidism, hypothyroidism, goiter/benign thyroid nodules, intellectual disability (IQ < 70), spontaneous abortion. We considered direct medical costs (i.e. resource use for the provision of healthcare services in the healthcare sector) and direct non-medical costs (i.e. travel costs to the clinic). We also assessed costs for productivity losses due to the absence of work.

All costs are reported in Euro (€) for the index year 2018. Resource use for medical consultations, laboratory tests and medication for the different diseases is based on guidelines of national and international expert associations, revised and adapted by clinical experts (17, 18, 19, 20, 21, 22, 23, 24, 25, 26). Dosage of medication was based on the WHO defined daily dose (27).

Inpatient costs were based on the ‘case flat rates’ (Fallpauschalen) of the German Diagnosis Related Groups System (G-DRG) (28) with the 2018 ‘federal base-case value’ (Bundesbasisfallwert) of € 3467.30 (29). G-DRGs are a classification system for a flat-rate reimbursement process in Germany. Patients (cases) are assigned to diagnosis-related case groups based on clinical data. The classification is usually determined by the type and severity of disease, and the services provided (e.g. surgeries and procedures which are assigned to ‘surgery and procedure codes’ (OPS-Codes)). The federal base-case value serves as a monetary value at federal level for calculating the DRG rates (28). For example, the reimbursement amount for a patient with goiter treated with thyroidectomy are calculated by assigning the patient to a specific G-DRG and a related OPS-code determining a specific factor and multiplying this factor with the federal base-case value.

Outpatient costs for doctor’s consultations, diagnostic procedures and laboratory tests were based on the ‘Uniform Value Scale of the Federal Association of Physicians under the Statutory Health Insurance’ (Einheitlicher Bewertungsmaßstab (EBM) der Kassenärztlichen Bundesvereinigung) (30) and the ‘statutes of medical fees’ (Gebührenordnung für Ärzte) (31) weighted according to the proportion of patients insured by statutory health insurances and private health insurances (32). The ‘Uniform Value Scale of the Federal Association of Physicians under the Statutory Health Insurance defines the content of reimbursable medical services and material costs, divided into general practitioner, specialist, and services and material costs reimbursable for both of them (30). The statutes of medical fees regulate the invoicing of medical services for private medical care in Germany not refundable by the statutory health insurances (e.g. for patients with private health care insurance) (31). Table 1 shows an example of the calculation of costs for a patient with hyperthyroidism. Costs for different services and materials which can be refunded by the statutory health insurances are listed in the ‘Uniform Value Scale of the Federal Association of Physicians, while costs which can be refunded by private health insurance are listed in the statutes of medical fees.

The costs for medications were evaluated according to the German reimbursement rules. Health insurances reimburse a fixed price for drugs of a specific category or the actual drug price, if it is lower than the fixed price (33). We calculated a weighted average of the five most frequently consumed drugs in each category (e.g. the category anti-thyroid medication) (34). The prices for medication were based on the data from the German pharmacy price schedule (Lauer Taxe) 2018 (35). We also considered out-of-pocket costs for patients according to the regulation of the statutory health insurance for additional costs. These regulations indicate that insured persons pay 10% of the selling price of a drug, at least € 5 but no more than € 10 (36). We considered a flat rate of € 10 per doctor’s visit or hospital stay for direct non-medical costs.

Indirect costs were based on productivity loss occurring due to the incapacity for work and the individual labor costs (i.e. gross wage rate and employer contributions to social insurance). The Federal Ministry of Health provides average days of absence due to illness for different International Classification of Diseases (ICD) -10 diagnoses according to health insurance data. We used the official numbers of the Federal Ministry of Health for average days of incapacity to work per case per year for the different IDDs to calculate productivity loss (37). Labor costs per day were calculated by multiplying the average working hours per day with average individual labor costs per hour (38). According to the German guidelines for economic analysis (12) we multiplied 80% of the average labor costs per day with the average days of incapacity to work per case for each IDD to receive the costs for productivity loss per year.

Costs for thyroid disorders in the year of diagnosis reflect the outpatient costs for doctor’s visits, including consultations and diagnostic tests. Additionally, costs for pharmaceuticals, scintigraphy, fine-needle biopsy, thyroidectomy, and radioiodine therapy are included for the different health conditions. We calculated costs according to different probabilities and assumptions with regard to proportions. For example, we assumed that a specific proportion of patients receive scintigraphy (19.6 percent) and fine-needle biopsy (1.4 percent) each year according to data from the literature (39) and expert opinion and added these costs to the total average costs per case for goiter or benign thyroid nodules.

We calculated a weighted mean of costs for different types of thyroidectomies ‘surgery and procedure codes’ (‘Operationen- und Prozedurenschlüssel (OPS-Codes)): 5-060, 5-061, 5-062, 5-063, 5-064) based on the proportional distribution of those surgeries (40). We calculated weighted mean costs according to a specific distribution of different radioiodine therapies (OPS-Codes: 8-531.00, 8-531.01, 8-531.10, 8-531.1, 8-531.20, 8-531.21) for goiter, benign thyroid nodules, and hyperthyroidism based on literature (41). For the treatment of hypothyroidism and hyperthyroidism, we calculated the annual costs for hormone replacement therapy and anti-thyroid medication, respectively.

The average costs per case of miscarriage include the costs for doctor’s visits, sonography, and proportionate costs for the medical and surgical management. We calculated average costs using prevalence data of the different management strategies from published literature (42, 43).

The annual costs for intellectual disability in Germany were available in the literature (44) for 2011. We transformed these costs in Euro 2018 using the overall consumer price index (CPI) for Germany (45).

We conducted an expert interview to estimate the resource utilization of an IDD prevention program including the iodine fortification of salt. We derived costs to the German government of administering and enforcing an iodine fortification program, costs for promotion campaigns and population information (every 2 years), additional costs for iodized salt for the consumer and costs for IDD monitoring (including a monitoring study every five years with 10,000 study participants). We calculated annual average cost per person for an IDD prevention program, total costs for IDD prevention and monitoring program in Germany and cases of the different IDDs and related treatments that need to be avoided for an IDD prevention program using universal salt iodization to be cost-saving.

Resource use for the different diseases, health conditions and treatments are summarized in Table 2. Table 1 shows an example of the calculation method for hyperthyroidism in the year of diagnosis.

**Table 1:** Example schematic for a cost calculation: hyperthyroidism in the year of diagnosis.

Uniform Value Scale (89.7% of population) (31)			Statutes of medical fees (10.3% of population) (32)		
Resource use		Costs in Euro 2018	Resource use	Costs in Euro 2018	
1st doctor’s visit					
One-time flat rate per insured person		24.14	Consultation	10.72	
Full-body examination		12.04	Full-body examination	34.86	
ECG		8.52	ECG	15.95	
2nd doctor’s visit					
Consultation flat rate		1.92	Symptom-related examination	10.72	
Sonography of the thyroid		9.06	Sonography of the thyroid	26.81	
			Blood sampling	4.19	
Laboratory		13.15	Laboratory	79.6	
3rd doctor’s visit					
Consultation flat rate		1.92	Symptom-related examination	10.72	
			Discussion of results	17.43	
Follow-up visit after 3 months					
Consultation flat rate		1.92	Symptom-related examination	10.72	
Sonography of the thyroid		9.06	Sonography of the thyroid	26.81	
			Blood sampling	4.19	
Laboratory		13.15	Laboratory	79.6	
Follow-up visit after 6–12 months					
Consultation flat rate		1.92	Symptom-related examination	10.72	
Sonography of the thyroid		9.06	Sonography of the thyroid	26.81	
			Blood sampling	4.19	
Laboratory		13.15	Laboratory	79.6	
SUM		119.01	SUM	453.64	
Weighted mean (0.897 × SUM Uniform Value Scale + 0.103 × SUM ‘Statutes of medical fees’) according to the proportion of patients insured by statutory health insurances and private health insurances (33):				153.58	
Direct non-medical costs (€ 10 per visit for transportation)				50	
Anti-thyroid medication		Sold defined daily dosis (DDD) in 2016 (Mio.) (28)	Proportion	Costs per DDD (Euro 2018) (36)	Additional costs for patients (37)
Thiamazol		26.1	0.58	0.17	0.05
Carbimazol		16.1	0.36	0.36	0.05
Natriumperchlorat		1.5	0.03	0.67	0.07
Propylthiouracil		1.1	0.02	0.29	0.05
Mean costs per DDD (Euro 2018) = proportion × (costs per DDD + additional costs for patients)					0.31
Costs per patient per year (Euro 2018) = 365 × mean costs per DDD					112.62
Total costs per case for hyperthyroidism in the year of diagnosis (Weighted mean Uniform Value Scale/Statutes of medical fees + direct non-medical costs + medication costs)					316.19
ICD 10-Code	Diagnosis	Absence of work in days per case year (38)	Total labor costs per day^a^	Estimation according to IQWIG guidelines (13) (80% of labor costs)	Monetary productivity loss per year per person with hyperthyroidism
E05	Hyperthyroidism	18.17	169.86	135.89	2469.13
Total costs per case for hyperthyroidism in the year of diagnosis including productivity loss (Weighted mean Uniform Value Scale/Statutes of medical fees + direct non-medical costs + medication costs + productivity loss)					2785.32

^a^Assuming 35.6 h of work per week and labor costs of 33.40 €/h (39).

ATM, anti-thyroid medication.

**Table 2:** Resource use and average days of incapacity to work for the different diseases, health conditions and treatments.

Disease or health condition	Resource use	Average days of incapacity to work
Hyperthyroidism in the year of diagnosis (without thyroidectomy/RAI) (35, 47)	1. Doctor’s appointment (1st)– Full-body consultation– ECG2. Doctor’s appointment (2nd) – Thyroid sonography – Blood sampling, laboratory tests3. Doctor’s appointment (3rd) – Consultation, discussion of results4. Doctor’s appointment (3-month follow-up) – Consultation, examination – Blood sampling, laboratory tests5. Doctor’s appointment (6- to 12-month follow-up) – Consultation, examination – Thyroid sonography – Blood sampling, laboratory tests6. Medication – Anti-thyroid medication for 9 months	18.17
Euthyroid health condition with ATM (individuals one year after diagnosis of hyperthyroidism) (35, 47)	1. Doctor’s appointment (follow-up) (one per year) – Consultation, examination – Thyroid sonography – Blood sampling, laboratory tests2. Medication – Hormone replacement therapy for 12 months	0
Hypothyroidism in the year of diagnosis (25, 35)	1. Doctor’s appointment (1st) – Full-body consultation – ECG2. Doctor’s appointment (2nd) – Thyroid sonography – Blood sampling, laboratory tests3. Doctor’s appointment (3rd) – Consultation, discussion of results4. Doctor’s appointment (3-month follow-up) – Consultation, examination – Blood sampling, laboratory tests5. Doctor’s appointment (6 –12 month follow-up) – Consultation, examination – Thyroid sonography – Blood sampling, laboratory tests6. Medication – Hormone replacement therapy for 9 months	17.11
Euthyroid health condition with HRT (individuals one year after diagnosis of hypothyroidism) (25, 35)	1. Doctor’s appointment (follow-up) (one per year) – Consultation, examination – Thyroid sonography – Blood sampling, laboratory tests2. Medication – Hormone replacement therapy for 12 months	0
Goiter/thyroid nodules (without thyroidectomy/RAI) (18, 19, 22, 48)	1. Doctor’s appointment (1st) – Full-body consultation – ECG2. Doctor’s appointment (2nd) – Thyroid sonography – Blood sampling, laboratory tests3. Doctor’s appointment (3rd) – Consultation, discussion of results4. Doctor’s appointment (3-month follow-up) – Consultation, examination – Blood sampling, laboratory tests5. Doctor’s appointment (6 –12 month follow-up) – Consultation, examination – Thyroid sonography – Blood sampling, laboratory tests6. Optional: scintigraphy and fine-needle biopsy	17.51
Spontaneous abortion (43, 44)	Option 1: Expectant care – Noninvasive assistance – Consultation of female genitalia – SonographyOption 2: Medical care – Noninvasive assistance – Sonography – Medication Option 3: Surgical care: First consultation – Noninvasive assistance – Consultation of female genitalia – SonographyOption 3: Surgical care: Surgery (OPS-Code 5-751) – Average period of hospitalization: 3 daysOption 3: Surgical care: Second consultation	9.31
Thyroidectomy (41)	Weighted mean of costs for different types of thyroidectomies OPS-Codes: 5-060: Incision in the area of the thyroid gland (2.7%) 5-061: Hemithyreoidectomy (28.2%) 5-062: Other partial thyroid gland resection (18.6%) 5-063: Thyroidectomy (50.2%) 5-064: Thyroid surgery via sternotomy (0.3%)	-
Radioiodine therapy (42)	Weighted mean of costs for different types of radioiodine therapies OPS-Codes: Radioiodine therapy up to 1,2 GBq I-131 8-531.00: without rh-TSH (64.6%) 8-531.01: with rh-TSH (4.3%) Radioiodine therapy from 1,2 to 5 GBq I-131 8-531.10: without rh-TSH (19.7%) 8-531.1: with rh-TSH (6.1%) Radioiodine therapy with 5 or more GBq I-131 8-531.20: without rh-TSH (3%) 8-531.21: with rh-TSH (2.3%)	-

ATM, anti-thyroid medication; GBq, gigabecquerel; HRT, hormone replacement therapy; I-131, radioisotope of iodine; IDD, iodine deficiency disorders; OPS, ‘German surgery and procedure code’ (Operations- und Prozedurenschlüssel); RAI, radioiodine therapy; rh-TSH, recombinant thyrotropin.

To calculate thresholds projecting how many IDD cases or related treatments would need to be avoided for an IDD prevention program to be cost-saving, we divided the total costs for an IDD prevention program in the German population by the costs for the different health conditions and related treatments.

## Results

Direct costs per case for the different IDDs ranged from 167 (euthyroid health condition with hormone replacement therapy) to € 14,202 (intellectual disability) per year. Including costs due to productivity loss, costs raged from 167 (euthyroid health condition with hormone replacement therapy) to € 17,497 (intellectual disability) per year. Costs for thyroidectomy and radioiodine therapy were € 4184 and € 3118, respectively. Direct costs for IDD diagnosis and treatment and costs including productivity loss are summarized in Table 3.

**Table 3:** Costs for IDD diagnosis and treatment.

Health condition or procedure	Annual average costs per case (Euro 2018)	Annual average costs per case including productivity loss	References for resource use	References for costs
Hyperthyroidism in the year of diagnosis (without thyroidectomy/RAI)	316	2785	IGES, 2017; AWMF, 2011	EBM, 2018; GOÄ, 2018; Lauer Fischer 2018
Euthyroid health condition with ATM	200	200	IGES, 2017	EBM, 2018; GOÄ, 2018; Lauer Fischer 2018
Hypothyroidism in the year of diagnosis	274	2599	Brenta, 2013; IGES, 2017	EBM, 2018; GOÄ, 2018; Lauer Fischer 2018
Euthyroid health condition with HRT	167	167	IGES, 2017	EBM, 2018; GOÄ, 2018; Lauer Fischer 2018
Goiter/thyroid nodules (without thyroidectomy/RAI)	211	2541	AWMF, 2015	EBM, 2018; GOÄ, 2018
Intellectual disability (IQ < 70)	14,202	17,497	Gustavsson, 2010	Gustavsson, 2010
	**Average one-time costs in Euro 2018**			
Spontaneous abortion	916	2181	Jurkovic, 2013; Trinder 2006	InEK, 2018; EBM, 2018; GOÄ, 2018
Thyroidectomy	4184		Maurer, 2015	InEK, 2018
Radioiodine therapy	3118		Hellwig, 2017	InEK, 2018

ATM, anti-thyroid medication; EBM, ‘Uniform Value Scale’ (Einheitlicher Bewertungsmaßstab); GOÄ, ‘Statutes of medical fees’ (Gebührenordnung für Ärzte); HRT, hormone replacement therapy; IDD, iodine deficiency disorders; InEK, ‘Institute for the reimbursement in hospitals’ (Institut für das Entgeltsystem im Krankenhaus); IQ, intelligence quotient; RAI, radioiodine therapy.

In the German population (around 82.17 million persons), total annual costs for implementing an IDD prevention program including iodine fortification of salt would amount to € 8,152,812 in the first year and € 8,002,812 annually in the years after implementation. Costs for an IDD prevention and monitoring program are summarized in Table 4.

**Table 4:** Estimated cost investments for an IDD prevention and monitoring program in Germany (expert estimation).

Type of resource use	Annual average costs per person in the German population in Euro 2018 (€)
Annual average per-person costs for salt iodization	0.09
Annual average per-person costs for IDD monitoring every 5 years	0.0187
Annual average per-person costs for IDD information campaign every 2 years	0.0037
One-time per-person public expenses for administration	0.0018
Total annual costs for the German population (first year)	8,152,812
Total annual costs for the German population (following years)	8,002,812

IDD, Iodine deficiency disorders.

To be cost-saving, the IDD prevention and monitoring program would need to prevent 26,000 cases of hyperthyroidism or 29,200 cases of hypothyroidism or 37,900 cases of goiter/thyroid nodules or 560 cases of intellectual disability or 8700 cases of spontaneous abortions per year. It would also be cost-saving if it could prevent 1900 thyroidectomies or 2600 radioiodine therapies per year. If an IDD prevention and monitoring could prevent a combination of different cases of IDDs, even less saved cases are needed of a single disorder or treatment for a cost-saving program.

## Discussion

We conducted a comprehensive cost analysis to evaluate costs for prevention, monitoring and treatment of IDDs in Germany, which provides important data for further health technology assessments. We also performed threshold analyses estimating how many cases of IDDs or related treatments would need to be prevented for an IDD prevention program to be cost-saving. To our knowledge, this is the first comprehensive and detailed cost analysis of IDD prevention, monitoring and treatment in a European country, including all potentially relevant consequences of iodine deficiency. Although there are few economic evaluations of IDD prevention programs (46, 47, 48), these results cannot be easily applied to the German healthcare context or interpreted from a German perspective due to the variations in availability of IDD prevention, different treatment approaches and varying resource utilizations and costs in the different health care systems.

A study conducted in Australia and New Zealand estimated the costs of preventing one person from having an urinary iodine concentration below 50 µg/L (100 µg/L) to be A$ 104.35 (A$ 1.81) per prevented case in Australia and NZ$ 15.30 (NZ$ 1.46) per prevented case in New Zealand and concluded that the costs appear small compared with the potential benefits (improved health, reduced health care costs and gains in productivity and GDP) (47). Another study in the UK evaluated the costs and benefits of iodine supplementation for pregnant women in a mildly to moderately iodine-deficient population by applying a decision-analytic model. They concluded that iodine supplementation is cost-saving in the UK context (48). Another health economic evaluation aimed to evaluate the cost-effectiveness of different alternatives for IDD elimination in Sikkim (India), including iodine fortification of salt, iodized oil injection program for high-risk groups and no preventive program. The cost per visible goiter person years, endemic cretinism, and IDD-attributable death were calculated in this analysis. The results of the analysis suggested that an iodized oil program might be less costly per averted case of IDD than the iodine fortification of salt in the state of Sikkim, India (46).

Kahaly *et al.* estimated the cost of thyroid disorders in Germany (7). They found that endemic iodine deficiency goiter causes economic costs of approximately 1 billion Euros per year. The per-patient costs for radioiodine therapy were estimated between 1530 and 1856 Euros depending on the type of hospital. Our results suggest even higher annual costs for the diagnosis and treatment of goiter. Assuming a goiter prevalence of about ten percent (49) (8.22 million cases) and about 72,000 thyroidectomies due to goiter (40), costs would amount up to more than 2 billion Euros per year. We also calculated higher costs for radioiodine therapy compared to the costs from the analysis conducted by Kahaly *et al*. However, methods used by Kahaly *et al.* for cost estimation are not completely clear. Additionally, Kahaly *et al.* did not calculate costs depended on treatment patterns and resource use for single diseases and health conditions. However, they concluded that ‘better prevention of iodine deficiency and its long-term consequences should effectively reduce direct as well as indirect costs and the overall economic impact of endemic goiter as the most important thyroid disease in Germany’ (7). This is in line with our conclusions.

In 1981, Germany introduced USI in a stepwise approach. Unfortunately, there are no data available on IDD prevalence and incidence before and after the introduction of USI in Germany. However, Denmark monitored the introduction of iodized salt in the country and published the results of the DanThyr study in various papers (39, 50, 51, 52). Before the implementation of an IDD prevention program in Germany in the year 1981, the population was moderately iodine deficient and the prevalence of goiter was 15% (53), similar to the prevalence of goiter in the moderately iodine-deficient region in the DanThyr study. Laurberg *et al.* (52) found a reduction of goiter from 17.6 to 10.9% within seven years. Assuming the same clinical effectiveness of USI in Germany, about 814.000 prevented goiter cases per year due to the introduction of salt iodization can be estimated. In this case, USI in Germany would already be cost-saving based on the results of our analysis.

The DanThyr study also analyzed ‘the utilization rate of surgery and radioiodine therapy for benign thyroid disorders before and after the introduction of iodization’ (51). Although the DanThyr study did not find a significant reduction of thyroidectomies, the authors found a decrease in radioiodine therapies of 12%. Our analysis for Germany showed high expected health-care expenditures for the treatment and management of IDDs due to thyroidectomies and radioiodine therapy. About 47,000 radioiodine therapies were performed in Germany in 2005 (54) and more than 50,000 were performed in 2014 (55). Unfortunately, there are no data available on radioiodine therapy in Germany before the introduction of iodized salt. We assume that there were even more radioiodine therapies in Germany before salt iodization. Assuming the same preventive effect of salt iodization in Germany as in the moderately iodine-deficient region in Denmark, such an IDD prevention program would prevent more than 6000 radioiodine therapies per year (based on a 12% reduction of 50,000 radioiodine therapies per year). Based on our calculations, the IDD prevention program would already be cost-saving if it was able to prevent 2600 radioiodine therapies per year. Therefore, assuming a combination of different effects of the IDD prevention program (e.g. prevention of goiter and prevention of radioiodine therapies), it is expected to save even more costs.

Based on our analysis, one of the most expensive components of the total costs were costs for inpatient care. Therefore, health care costs could also be reduced by a reduction of treatment costs or length of hospitalization. For example, about 90,000 thyroidectomies are performed annually in Germany. More than 80% of these procedures involve the resection of euthyroid goiter or benign thyroid nodules, 15–20% of the surgeries are performed in patients with hyperthyroidism and about one percent of the interventions are performed in patients with thyroid carcinoma (40). Ongoing discussions in Germany include criticism on the high frequency of total thyroidectomies (56, 57). A discussion on and adaption of the current treatment guidelines could also potentially reduce costs in the treatment of IDDs in addition to the effect of an IDD prevention program.

Our analysis suggests that an IDD prevention and monitoring program could be cost-effective or even cost-saving, if a combination of IDDs and related treatments could be prevented by the USI. The results of the DanThyr study confirm the reductive effect of USI on IDDs and related health conditions. However, a comprehensive health economic evaluation of an IDD prevention program would be needed to assess the full effects of a population-wide IDD prevention program on the population’s life expectancy and quality of life including all relevant benefits, risks and costs of an IDD prevention and monitoring program. Additionally, current and future populations would need to be considered to capture the full effect of an IDD prevention program on the currently alive population as well as the partial effects accumulated by future generations, which is beyond the scope of our cost analysis.

Furthermore, benefits and harms of a population-wide IDD prevention program should be carefully evaluated and weighted against each other. There are several iodine fortification and supplementation strategies worldwide (2) and monitoring studies for their assessment (58). Overall, iodine fortification of salt seems to reduce mean thyroid size and the prevalence of diffuse goiter at all ages a few years after the introduction of iodized salt (59, 60, 61, 62). Studies also demonstrated that iodine prophylaxis before or during early pregnancy eliminates new cases of developmental delay due to iodine deficiency and increases developmental scores in children in moderate and severely iodine-deficient regions (59, 63). However, salt iodization seems to have no effect on overt hypothyroidism (59, 64, 65). In some areas with iodine fortification, an increase of subclinical hypothyroidism can be observed which may be reversible, but a reduction of the iodine intake might be required to normalize thyroid function (66). Further research in this field could provide more information on the underlying mechanisms and the long-term outcomes for patients with iodine-induced subclinical hypothyroidism. Also, the connection between salt iodization and hyperthyroidism has not been finally resolved yet (6, 59, 67). Some research in this field suggests that a monitored implementation of iodine prophylaxis may only result in a transient increase of the incidence of hyperthyroidism (6). Iodine deficiency during pregnancy is not only associated with congenital hypothyroidism of the newborn but may also cause other adverse pregnancy outcomes (68). Severe iodine deficiency is associated with miscarriage, stillbirth, neonatal mortality, low birth weight and poor fetal growth (69, 70). However, a recent study showed that mild to moderate iodine deficiency in developed countries does not lead to an increased risk of pregnancy loss (71). Further research is required in this field for future decision making. Since the adverse effects of IDD prevention programs could also outweigh the benefits, there is a need for a comprehensive synthesis of the current evidence related to the effectiveness and safety of IDD prevention programs.

Our study has several limitations. The data on treatment patterns and resource use in the German health care setting are based on guidelines of national and international expert associations. However, real-world treatment patterns and resource use may differ from recommended patient pathways. This could lead to an under- or overestimation of costs. For a detailed resource analysis, patient pathways would need to be monitored in a systematic way (e.g. in a prospective study design).

Cost data collected through prospective data collection processes (e.g. a cost analysis during a randomized controlled trial) might also result in more reliable cost estimations. We could not analyze the detailed costs of every single resource used in the prevention, diagnosis and treatment of the different IDDs. We used average amounts of different resources such as flat rates, mean hospital days or mean days of illness with absence from work. Costs could be over- or underestimated depending on the actual use of the different resources per case. However, resource utilization was revised and adapted by German clinical experts and compared to diagnostic and therapeutic procedures conducted in clinical trials and prospective cohort studies.

We also used expert estimations for different proportions and probabilities if we could not obtain these data from any study or registry (e.g. the proportion of patients with goiter or benign thyroid nodules receiving a scintigraphy). Therefore, the cost-analysis should be updated as soon as those data are available.

For the treatment and care of patients with intellectual disability, we used data collected for a cost analysis in the Netherlands and transformed them into 2018 Euro. However, treatment and care for individuals with intellectual disability in the Netherlands may differ from those in Germany. Therefore, costs might be under- or overestimated, depending on the differences in resource utilization in both countries. However, the study used a top–down approach with a health-care perspective similar to our methods.

Our threshold analyses only analyzed the effectiveness of an IDD prevention program on single outcomes such as the incidence of goiter or thyroidectomies. However, we expect a simultaneous effect of an IDD prevention program on multiple diseases, treatments and outcomes. Therefore, the estimated thresholds for each single disease or treatment are just upper limits and the real thresholds for an IDD prevention program to be cost-saving should be much lower.

The threshold analyses indicate that the benefits of an IDD prevention program outweigh the harms of such a program based on current literature. However, there are also studies questioning the efficacy of IDD prevention programs (59, 71) or even reporting harms due to iodine fortification on a population level (65, 67). Therefore, future research should focus on comprehensive and country-specific benefit-risk assessments. For a full health economic evaluation of an IDD prevention and monitoring program, other important aspects need to be considered in future research (e.g. the currently alive German population plus their children born during a specified analytic time horizon, data on the epidemiology of the different IDDs in different age groups, effectiveness data of an IDD prevention program). Outcomes such as (quality-adjusted) life-years gained and incremental cost-effectiveness and cost-utility ratios (ICER, ICUR) should be estimated. Decision-analytic modeling is an approach that allows for synthesizing current evidence to support decision making under uncertainty by including all available interventions (e.g. therapeutic or preventive strategies) and all possible health consequences of the different alternatives and compare these interventions with regard to the outcome of interest (10, 11). Decision analysis could be used for a full economic evaluation of the long-term effectiveness and cost-effectiveness of an IDD prevention and monitoring program. Our cost analysis provides excellent data and model input parameters for such decision analyses.

## Conclusion

Based on our cost analysis, an IDD prevention program potentially saves costs, if a specific minimum amount of iodine deficiency-related health conditions and/or treatments per year could be avoided by the prevention program and the overall benefits of such a program would outweigh adverse outcomes caused by such a program (e.g. additional cases of subclinical hypothyroidism or hyperthyroidism). However, in order to recommend the implementation of an IDD prevention program, epidemiological and long-term effectiveness, as well as safety data, need to be considered in a full health economic evaluation based on a decision-analytic model. The results of our cost analysis can be included in such decision analyses to evaluate the overall long-term effectiveness, benefit-harm balance and cost-effectiveness of an IDD prevention program.

## Declaration of interest

The authors declare that there is no conflict of interest that could be perceived as prejudicing the impartiality of the research reported.

## Funding

This work was supported by EUthyroid. The project has received funding from the European Union’s Horizon 2020 research and innovation program under grant agreement number 634453.
